# The influence of job burnout on the attention ability of army soldiers and officers: Evidence from ERP

**DOI:** 10.3389/fnins.2022.992537

**Published:** 2022-11-07

**Authors:** Lin Wu, Yanfeng Chen, Xufeng Liu, Peng Fang, Tingwei Feng, Kewei Sun, Lei Ren, Wei Liang, Huijie Lu, Xinxin Lin, Yijun Li, Lingling Wang, Chenxi Li, Tian Zhang, Chunping Ni, Shengjun Wu

**Affiliations:** ^1^Department of Military Medical Psychology, Air Force Medical University, Xi’an, China; ^2^Nursing School, Air Force Medical University, Xi’an, China

**Keywords:** job burnout, attention, event-related potential (ERP), P300, soldiers and officers

## Abstract

Job burnout is one of the most widespread mental problems in today’s society and seriously affects the mental health and combat effectiveness of soldiers and officers. Herein, the effect of burnout on individual attention is studied from the perspective of neuroelectrophysiology. A total of 1,155 army soldiers and officers were included in this investigation and completed the Job Burnout Scale for Military Personnel. A total of 42 soldiers and officers were randomly selected from those with and without burnout to participate in an event-related potential (ERP) study using a visual oddball task. The characteristics of visual P3a and P3b at Fz, FCz, Cz, CPz, and Pz were recorded and analyzed by repeated-measures analysis of variance (ANOVA). *P* < 0.05 was the criterion for a significant difference. The total average score on the Job Burnout Scale for Military Personnel among the participants was 0.74 ± 0.46, and the detection rate of job burnout was 29.85%. In the Oddball task, the average number of target stimuli counted in the burnout group was lower than that in the control group, but no significant difference was found. For P3a, the Fz, FCz, Cz, CPz, and Pz amplitudes in the burnout group were significantly lower than those in the control group. The average amplitude of P3a evoked in the central parietal area was larger than that in the prefrontal area. For P3b, the amplitudes of the five electrodes in the burnout group were significantly lower than those in the control group. The average amplitude of P3b evoked in the parietal region was larger than those in the prefrontal and central parietal regions. A certain degree of job burnout is evident in army soldiers and officers. The voluntary attention and involuntary attention of individuals with burnout are both affected to some extent, as reflected by the lower amplitudes of P3a and P3b. The results suggest that P3a and P3b can be used as indicators to monitor cognitive neural function in soldiers and officers with burnout and can also be used as references for evaluating the effects of cognitive training and screening methods. In this study, ERP was used to research the attention ability of soldiers and officers with job burnout, and related issues were discussed from the aspects of the burnout results, behavioral results, ERP results, compensation effect of cognitive resources, application in the military field, limitations, and prospects.

## Introduction

In 2019, the World Health Organization (WHO) proposed that “job burnout” should be included in the 11th Edition of the International Classification of Disease (ICD-11), indicating that job burnout has become an important indicator of mental health worldwide (World Health Organization [WHO], 2019). Job burnout is a concept that was first proposed in 1974 by the American clinical psychologist Freudenberger. It refers to a series of multidimensional psychological symptoms related to work and delayed reactions caused by work and interpersonal pressure (Freudenberger, 1986; Maslach and Leiter, 2010). It is a state of physical and mental exhaustion, with effects on physiology, emotions, cognition, behavior, etc., and is also known as burnout syndrome (BS) (Maslach et al., 2001; Mauranges, 2018). It also has a negative influence on physical health, subjective well-being, work performance, and other aspects (Tims et al., 2013; Zarafshan et al., 2013).

In peacetime, soldiers and officers are always ready to fight, undertake many urgent and dangerous tasks, undergo extensive training, and live in difficult conditions, where the environment is relatively closed and harsh. New combat styles, territorial expansion, all geographies, all weather conditions, high-damage combat, and other characteristics of modern wars serve as major tests of the mental health of military personnel. As an important aspect of mental health, job burnout has become an important factor leading to a reduction in loyalty, affecting army morale and restricting improvements in combat effectiveness (Campbell and Nobel, 2009; Adler et al., 2017; Frone and Blais, 2019). The incidence of job burnout among troops, armed police, and highly educated military personnel is increasing (Sun et al., 2013a). The job burnout scores for military personnel in special working environments are also significantly higher than that in the urban (Tao et al., 2014). A research by Chinese scholars indicates that job burnout among armed police officers is serious; the detection rates of emotional exhaustion, depersonalization, and reduced professional efficacy are 11.9, 42.8, and 36.7%. The detection rates of mild, moderate, and severe burnout are 54.4, 34.7, and 2.2%, respectively (Huang, 2012). These rates are higher than those for other general staff. Ahola et al. (2006) reported that only approximately 25% of the general population has been observed to suffer from job burnout, and 2% of cases are estimated to be severe. Burnout percentages are reported to range from 4.3 to 25% based on data from the 6th European Working Conditions Survey. Based on the results of Chinese and foreign studies, some scholars have proposed that the main factors affecting job burnout among military personnel are demographic factors, job characteristics, organizational characteristics, and individual characteristics (Chen et al., 2019), including occupational stress, social support, personality traits, social strategies, group culture, and personal participation in the working group (Sun et al., 2013b).

Attention is an important field of cognitive psychology and is considered one of the cognitive abilities that occur relatively early after an event. As a psychological phenomenon, attention is widely involved in emotional, decision-making, memory, and other psychological processes, which ensures that people have a clearer understanding of things, a more accurate response, and controllable behavior (Ben et al., 2009; Grossberg, 2021). Attention has the characteristics of directivity and concentration. Directivity determines to which object mental activity or consciousness is directed, and concentration refers to the intensity of focus on an object to ensure the selection of effective information and the effective handling of problems (Carlisle, 2019). According to the function of attention, attention ability can be divided into selective attention, persistent attention and distributive attention; according to whether a goal has been set and the degree of will or effort, attention can be divided into voluntary attention and involuntary attention. Voluntary attention refers to the goal-directed cognitive processing that individuals need to make subjective efforts and is determined by the relevant task at hand, while involuntary attention refers to an orientation response in which attention resources are passively invoked when the sudden stimuli are strong enough (Landau et al., 2012; Giraudet et al., 2015; Purokayastha et al., 2021). In this study, we discuss the voluntary and involuntary attention of burnout soldiers and officers.

Event-related potentials (ERPs) represent an important method for studying cognitive activities. It has a high time resolution ratio and the characteristic of time locking, reflecting the brain’s response under specific tasks and events of interest. ERPs can provide information about how the human brain processes signals and prepares to take action; therefore, it has considerable advantages in revealing the temporal process of cognition, providing a complementary perspective and approach for the study of advanced cognitive neural function (Herrmann and Knight, 2001; Helfrich and Knight, 2019). P300 is considered the first endogenous component of ERPs and is a positive evoked potential approximately 300 ms after a stimulus is presented (Soltani and Knight, 2000). P300 has been confirmed to be related to many activities, such as attention, memory, and inhibition, reflecting the characteristics of the brain in the process of cognitive activities; thus, it is also called a cognitive potential (e.g., P1, N1, P2, N2, and P3) rather than an early evoked potential of sensory processing (Polich, 2007; Bravermanová et al., 2018). Isreal et al. (1980) believe that the brain must invest attention resources to identify and evaluate stimuli, and P300 can reflect the brain’s attention process. By changing the stimulus paradigm, sequence, frequency, interval, and other parameters, P300 is evidently not a single component but can be divided into two subcomponents that occur sequentially in time and represent different attention types and information processing procedures, namely, P3a and P3b. P3a, also known as early P300, is a positive cortical discharge approximately 200–280 ms after stimuli presentation and has a short latency. P3a is believed to mainly be induced by novel stimuli, representing the perception stage after a stimulus occurs (Polich, 2007). Compared with that of P3a, the latency of P3b is longer (310–380 ms). P3b is thought to be mainly induced by target stimuli, representing the stage of response to stimuli (Polich, 2007). It should be noted that in this study we are talking about visual P3a and P3b.

Job burnout is characterized by emotional exhaustion, depersonalization, and reduced professional efficiency (Maslach et al., 2001; Maslach and Leiter, 2016). A large number of studies have found that job burnout has a long-term impact on work ability, health status, and interpersonal relationships (Rodrigues et al., 2018; Roy, 2018; Laccourreye and Lisan, 2019; Tavella et al., 2021). At the same time, there is no doubt that job burnout damages attention and other kinds of cognitive functions. Research shows that individuals with job burnout experience more cognitive dysfunction, which is manifested in the decline of working memory, attention ability, executive function, and information processing ability (Cahn and Polich, 2006; Eskildsen et al., 2015; Gajewski et al., 2017a; Gudi-Mindermann et al., 2020; Schreiner and Staudigl, 2020). Jonsdottir et al. (2013) also found that individuals with job burnout performed poorly concerning attention span, working memory, learning, and situational memory. Sokka et al. (2017) also reported that job burnout affects individuals’ ability to switch tasks. As for the influence of job burnout on attention ability, many research works using electroencephalogram indicate that P300 amplitude is related to the allocation of attention resources (Ladouce et al., 2019). The amplitude of P300 decreases when an individual has impaired attention and difficulty concentrating or when a difficult task exceeds the adaptation range, resulting in attentional resource separation (Pergher et al., 2019). Van Luijtelaar et al. (2010) analyzed ERPs in patients with burnout compared to healthy controls. The burnout patients who were clinically diagnosed with burnout syndrome showed reduced P300 (P3b) amplitude, lower alpha peak frequency, and reduced beta power in an auditory Oddball task. Golonka et al. (2018) conducted ERP research using two experimental procedures—the Go/NoGo Task and the Doors Task—on employees who were screened by the Maslach Burnout Inventory – General Survey (MBI-GS). The decrease in P300 amplitude in the job burnout group revealed limited attentional resources with further processing of conflicting information. Sokka et al. (2014) researched the electrophysiological correlates of automatic sound change detection and involuntary attention in job burnout using P3a of ERP. They found an attention capture tendency in individuals with job burnout who are faster with negative information but slower with positive information compared to people in the control group. It can be seen that compared with the general population, individuals suffering from job burnout exhibit different characteristics of attention function (Van Dinteren et al., 2014).

Meanwhile, metabolomics and brain imaging also provide evidence for cognitive decline caused by job burnout. Belanoff et al. (2001) proposed that job burnout may result from long-term chronic stress exposure; glucocorticoids produced under long-term stress may enter the prefrontal cortex and destroy the neuronal structure of the brain. Marin et al. (2011) found that the right dorsolateral prefrontal cortex (dlPFC) was associated with a decline in the blood oxygen level dependence (BOLD) effect in the middle frontal gyrus of individuals with high levels of burnout. Blix’s study (Blix et al., 2013) indicated that the gray matter in the anterior cingulate gyrus and dlPFC in the caudate nucleus decreased significantly and connectivity changed also in individuals with higher levels of burnout. Since the prefrontal cortex is considered the most important brain area involved in cognitive activities, the structural and functional damage of the prefrontal cortex in burnout individuals directly affects the cognitive process and leads to declining task performance.

To date, research on the influence of job burnout on the cognitive function of military personnel has been limited. Military soldiers and officers are the group that performs special tasks, and the study of military personnel job burnout has special and important significance; however, traditional behavioral research still utilizes external indicators such as reaction time and accuracy, which have limitations.

In this study, ERP was used to study the attention ability of military soldiers and officers with burnout, and we explored the neuroelectrophysiological characteristics of the influence of burnout on attention. The hypothesis is that job burnout leads to a reduction in the attention resources of soldiers and officers. The voluntary and involuntary attention of burnout individuals is damaged to a certain extent, which is manifested by the decrease in P3a and P3b amplitudes in the frontal lobe, parietal lobe, etc. The results of this study provide the basis for the selection of reference indexes for monitoring, evaluating, and guiding interventions for military soldiers and officers with burnout.

## Participants and methods

### Participants

Using cluster sampling, the soldiers and officers of a land force unit who were not performing tasks were selected as the participants. The researcher conducted the overall measurements with the company as the unit. The inclusion criteria were as follows: (a) no use of psychoactive and anesthetic drugs and active substances within 1 week of the study and (b) individuals who signed informed consent after receiving information regarding the content of this experiment and agreed to participate in this study. The exclusion criteria were as follows: (a) individuals with a history of craniocerebral trauma and mental disorders and (b) individuals who were unwilling to cooperate. All the participants were informed of the purpose, precautions of the experiment and signed an informed consent form. The participants’ personal information (including name), the results of the scale, and whether to participate in the ERP test were only known to the experimenters and themselves but not visible to others to eliminate the participants’ doubts and ensure the data were real and valid. This study was approved by the Clinical Trial Ethics Committee of the First Affiliated Hospital of Air Force Medical University and is registered at the Chinese Clinical Trial Registry with the registration number CHiCTR1800019761.

The self-compiled General Information Questionnaire was used to collect general demographic information about the participants (gender, ethnicity, only child or not, marital status, major, age, education level, and identity). A total of 1,155 copies of the questionnaires were distributed, and 1,062 valid questionnaires were retrieved, for an effective recovery rate of 91.9%. All the participants were male and between 17 and 45 years of age, with an average age of 22.29 ± 3.22 years. The general demographic data are provided in Table 1.

**TABLE 1:** General demographic data (*n* = 1062).

Item	Number of subjects (n)	Percentage (%)	Item	Number of subjects (n)	Percentage (%)
**Nationality**			**Age**		
Han	1016	95.67	<20 years	370	34.84
Minorities	46	4.33	20–30 years	665	62.62
**Only child**			≥30 years	27	2.54
Yes	440	41.43	**Education level**		
No	622	58.57	Junior high school and below	82	7.72
**Marriage status**			High school or technical secondary school	630	59.32
Unmarried	969	91.24	Junior college and college	350	32.96
Married	93	8.76	**Identity category**		
**Professional status**			Military officer	59	5.56
Combat	542	51.04	Non-commissioned officer	362	34.09
Technology	446	42.00	Conscripts	641	60.35
Guarantee	74	6.96			

### Methods

#### Research tools

The Job Burnout Scale for Military Personnel compiled by Zhang (2011) was used. The scale has a total of 35 items divided into five dimensions, i.e., sense of accomplishment, somatization, self-evaluation, interpersonal relationships, demotivation, and one masking factor. Each item is scored from 0 to 3: “Never,” 0; “Occasionally,” 1 point, “Often,” 2 points; and “Always,” 3 points; items 6, 8, 17, 25, 28, and 31 are reverse scored. The scores for each item are added together to obtain the total score. The higher the score is, the more severe the degree of job burnout. The Cronbach’s α coefficient for the scale is 0.917, and the split-half reliability is 0.920. This scale is significantly correlated with the Chinese Maslach Burnout Inventory (CMBI), with a correlation coefficient of 0.070–0.725 (*P* < 0.01) (Zhang and Zhang, 2010), indicating that the Job Burnout Scale for Military Personnel has good reliability and validity and reaches the psychometric standard. This scale has been confirmed and widely used by many scholars (Dong et al., 2014; Chen et al., 2019; Wang et al., 2020). The Cronbach’s α coefficient of this scale was 0.920 in this study.

The oddball paradigm is considered to be a classic paradigm that can induce P300. In the traditional double-stimuli visual Oddball paradigm, participants are randomly presented with the target stimuli and standard stimuli. They are asked to respond to the target stimuli and ignore the standard stimuli. The occurrence rate of the target stimuli was far less than that of the standard stimuli. Snyder and Hillyard (1976) added novel stimuli to the target and standard stimuli sequence. They did not inform participants of the novel stimuli before the experiment and asked participants only to respond to the target stimuli as usual. This study adopted the three-stimuli visual oddball paradigm adjusted by Snyder, including target stimuli, standard stimuli, and novel stimuli. The participants’ active attention to target stimuli reflected the process of voluntary attention, and the passive orientation to novel stimuli reflected the process of involuntary attention. The three-stimuli Oddball paradigm can skillfully separate the two forms and processes of attention mentioned above at the same time (Huang et al., 2015).

### Experimental equipment

An electroencephalogram (EEG) was performed with a Neuroscan SynAmps 2 amplifier and acquisition system. EEG recordings were performed using a Neuroscan 32-lead Ag/AgCl electrode cap (32 single-lead EEG). The international 10–20 system was used. The reference electrodes A1 and A2 were located on both sides of the mastoid process. The two electrodes recorded the horizontal and vertical electrooculogram (EOG). Vertical EOG (VEOG) was recorded by electrodes located on the inferior and superior orbits of the left eye, and horizontal EOG (HEOG) was recorded by electrodes placed 1 cm lateral to both eyes. The collection electrodes were FP1, FP2, F3, F4, F7, F8, Fz, FC4, FC7, FC8, FCz, C3, C4, C7, C8, Cz, CP3, CP4, CP7, CP8, CPz, P3, P4, P7, P8, Pz, O1, O2, Oz, VEOG, and HEOG. In the experiment, the impedance of each electrode point was below 5 kΩ, the high-pass was set to 0.01 Hz, the low-pass was set to 100 Hz, the sampling rate was 1000 Hz, and the reference electrode was A2.

### Experimental process

The soldiers and officers selected by cluster sampling completed the Job Burnout Scale for Military Personnel. A total average score ≥1 on the Job Burnout Scale for Military Personnel indicated a certain degree of job burnout, not that the participants could be clinically diagnosed with job burnout.

Limited by the experimental conditions and military tasks, a random number table was used to select 21 individuals with (total average score of ≥1) and without burnout (a total of 42 people) to be included in the ERP study [$M=2\,\left[1+\left(K-1\right)\,\rho \right]\,\frac{\sigma^{2}\,{\left(Z_{1-\alpha \,/\,2}+Z_{1-\beta }\right)}^{2}}{K\,\delta^{2}}$ was used to estimate the sample size (Chang and Chang, 2012)].

**FIGURE 1:**
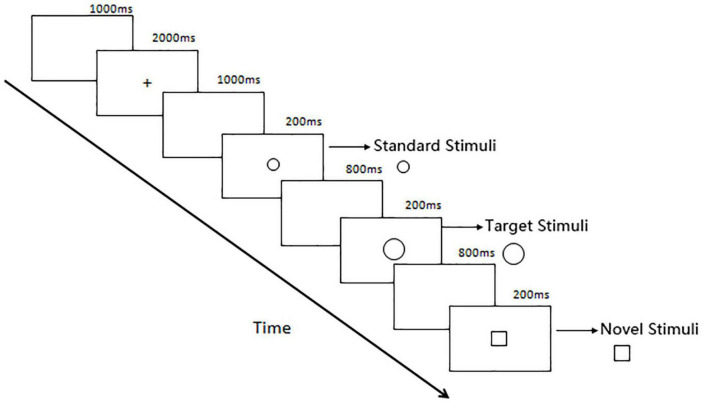
Flow chart of the oddball experiment.

The participants underwent ERP in a quiet, dark room. The background for the visual stimuli was a black blank screen (17-inch computer screen). The three-stimuli visual oddball paradigm was adopted for the ERP experiment, and the experimental materials were presented in a random manner through E-prime 3.0 software on the computer. The experiment was divided into one exercise block and four formal experimental blocks. The exercise block included 38 trials, and each formal experimental block included 80 trials: 12 trials for the target stimuli (a circle with a larger diameter), 56 trials for the standard stimuli (a circle with a smaller diameter), and 12 trials for the novel stimuli (squares). Participants could press a button to rest between each block to eliminate fatigue. In each part of the experiment, a blank screen was first presented for 1000 ms, followed by a “+” fixation point (font size, 28) in the center of the screen for 2000 ms and then a blank screen for 1000 ms; subsequently, the formal test was started. The presentation time for the stimulus image in each trial was 200 ms, and then a blank screen was displayed for 800 ms. The participants were asked to count the target stimulus pictures, verbally respond at the end of each test section, and ignore stimuli other than the target stimuli. The experimental procedure is shown in Figure 1. During the experiment, two participants who could not complete the test were excluded; ultimately, 19 participants were included in the burnout group, and 21 participants were included in the control group.

### Event-related potential data analysis

The ERP data were analyzed using MATLAB (2013b) software and the ERPLAB toolkit (Lopez-Calderon and Luck, 2014; Wen et al., 2016). After bad segments were manually removed, the following data processing operations were completed in sequence:

Filtering: based on previous research experience and experimental needs, a 1–40 Hz bandpass filter was selected to filter out the very low-frequency and high-frequency ineffective components;
  Epoch creation: create an event list, assign bins, and segment them into epochs using a time window of 1000 ms, ranging from 200 ms prestimulus to 800 ms poststimulus. Segments with wave amplitudes exceeding ±100 μV were removed;
  Removal of artifacts: the independent component correlation algorithm (ICA) was used for segmented data to eliminate artifacts such as EOG, saccades, and electromyography (EMG), as well as other noise interference;
  Baseline correction: baseline correction was performed using the prestimulus interval (−200 to 0 ms).
  Superimposed average: the target stimuli, standard stimuli, and novel stimuli were superimposed and averaged. We take −200∼0 ms as the baseline, use MATLAB code to analyze the ERP data, and then draw the waveform diagrams of the two groups (the burnout group and the control group) at the five electrode points (Fz, FCz, Cz, CPz, and Pz) on the midline of the brain. Use the Find function to find the point (300–450 ms) with the largest value on the curve on the vertical axis y. The difference between this value and the baseline is the peak of the P3a or P3b component.

### Statistical analysis

SPSS 22.0 software was used for statistical analyses. Descriptive statistical analysis, the *t*-test, and repeated measures analysis of variance (ANOVA) were used. For the P3a and P3b components, five electrode sites (Fz, FCz, Cz, CPz, and Pz) in the central frontal region and the central parietal region were selected. The amplitudes at the five electrode sites on the midline of the brain mentioned above were averaged to obtain the overall average amplitudes of P3a and P3b of the burnout group and the control group, respectively. Subsequently, the amplitudes of each group on the above five electrode sites were analyzed by ANOVA according to two groups (burnout group and control group) × 5 electrode sites (Fz, FCz, Cz, CPz, and Pz), main effect and interaction effect tests were performed. Further simple effect analysis was performed for the results with significant interaction effects. The data that did not satisfy the sphericity test were adjusted using the Greenhouse–Geisser method. We used $\bar{\text{x}}$ ± SE for data recording and *P* < 0.05 as the criterion for a significant difference.

## Results

### Demographic information, burnout score, and behavior task results

In this study, 1,155 copies of the Job Burnout Scale for Military Personnel were distributed, and 1,062 valid questionnaires were retrieved for an effective recovery rate of 91.9%. The total average job burnout score among military personnel was 0.74 ± 0.46, and 317 individuals had an average job burnout score ≥1, accounting for 29.85% of the total sample. Among the five dimensions, the self-evaluation score was relatively high, while the interpersonal relationship score was relatively low. The difference in burnout scores between the burnout group and control group was statistically significant (1.71 ± 0.19 vs. 0.80 ± 0.36, *t* = 9.912, *P* < 0.001). The results are shown in Table 2.

**TABLE 2:** Job burnout scores of military soldiers and officers (*n* = 1062, $\bar{\text{x}}$ ± s).

Dimension	Average
Sense of accomplishment	0.71 ± 0.50
Somatization	0.63 ± 0.52
Self-evaluation	1.12 ± 0.76
Interpersonal relationship	0.51 ± 0.53
Demotivation	0.72 ± 0.57
Total average job burnout score	0.74 ± 0.46

No significant difference in age (21.63 ± 2.27 vs. 21.71 ± 2.05, *t* = −0.121, *P* = 0.904), marital status (χ^2^ = 0.005, *P* = 0.942), or education level (χ^2^ = 0.478, *P* = 0.787) was found between the two groups; therefore, the sociodemographic factors were basically matched in the above aspects. The results are shown in Table 3.

**TABLE 3:** General information of ERP participants (*n* = 40).

Item	Burnout group (*n* = 19)	Control group (*n* = 21)	*P*
**Age**	21.63 ± 2.27	21.71 ± 2.05	>0.05
**Marriage status**			
Unmarried	18	20	>0.05
Married	1	1	
**Education level**			
Junior high school and below	2	3	>0.05
High school or technical secondary school	12	11	
Junior college and college	5	7	

In the Oddball task, the average number of target stimuli counted by the participants in the burnout group was lower than that in the control group, but no significant difference was noted between the two groups (11.47 ± 0.23 vs. 11.64 ± 0.34, *t* = −1.811, *P* = 0.078).

### Event-related potential results

The P300 components of the burnout group and control group were analyzed, and brain topography was mapped. Because P3a and P3b are located in the central frontal and central parietal regions, only five electrode sites in the central frontal and central parietal regions were analyzed, that is, Fz, FCz, Cz, CPz, and Pz. It is worth noting that in this study, the burnout group includes 19 participants and the control group includes 21 participants. Each participant has 4 formal blocks, and each block contains 80 trials. The burnout group has a total of 19 * 80 * 4 = 6080 trials, and the control group has a total of 21 * 80 * 4 = 6720 trials. Although some invalid trials (including ECG, EOG, EMG, and other artifacts) were deleted in the both burnout group and the control group, the numbers of trials retained in the two groups were still large, meeting the requirements for analysis.

### Comparison of the average amplitude of P3a between the burnout group and control group

The descriptive statistics of the average P3a amplitude at each electrode site in the burnout group and control group are shown in Table 4.

**TABLE 4:** Description of the average P3a amplitudes in the burnout group and control group ($\bar{\text{x}}$ ± SE, μV).

Electrode site	Burnout group (*n* = 19)	Control group (*n* = 21)
Fz	6.50 ± 0.93	9.51 ± 0.89
FCz	7.31 ± 0.89	10.80 ± 0.85
Cz	8.24 ± 0.81	10.77 ± 0.77
CPz	9.46 ± 0.80	10.90 ± 0.76
Pz	9.41 ± 0.74	10.02 ± 0.71

The repeated-measures ANOVA results indicated that the main effect between groups was significant, *F*(1, 38) = 4.254, *P* = 0.046, η^2^ = 0.11, and that the average P3a amplitude of the burnout group was significantly lower than that of the control group, i.e., 8.19 ± 0.78 and 10.40 ± 0.74 μV, respectively.

The main effect of the electrode was significant, *F*(4, 152) = 12.070, *P* < 0.001, η^2^ = 0.241, and the average amplitude of P3a induced at the electrode sites in the central parietal region was greater than that in the prefrontal region. The average amplitudes of P3a at Fz, FCz, Cz, CPz, and Pz were 8.00 ± 0.64, 9.06 ± 0.62, 9.51 ± 0.56, 10.18 ± 0.55, and 9.71 ± 0.51 μV, respectively.

The interaction effect between electrode and group was significant, *F*(4, 152) = 6.139, *P* = 0.009, η^2^ = 0.139. Further simple effect analysis indicated that in the burnout group, the average amplitude of P3a at Fz was smaller than that at FCz, Cz, CPz, and Pz (Fz < FCz < Cz < CPz < Pz), with significant differences (*P* = 0.007, *P* < 0.001, *P* < 0.001, *P* < 0.001); the average amplitude of P3a at FCz was smaller than that at Cz, CPz, and Pz (FCz < Cz < CPz < Pz), with significant differences (*P* = 0.002, *P* < 0.001, *P* = 0.003); the average amplitude of P3a at Cz was smaller than that at CPz and Pz (Cz < CPz < Pz), with significant differences (*P* < 0.001, *P* = 0.020); and the average amplitude of P3a at CPz and Pz was not significantly different (*P* = 0.840). In the control group, the average amplitude of P3a at Fz was smaller than that at FCz, Cz, and CPz (Fz < FCz < Cz < CPz), with significant differences (*P* < 0.001, *P* = 0.003, *P* = 0.024); the average amplitudes of P3a at FCz, Cz, CPz, and Pz were not significantly different (*P* = 0.909, *P* = 0.841, *P* = 0.220); the average P3a amplitudes at Cz, CPz, and Pz were not significantly different (*P* = 0.647, *P* = 0.109); and the average amplitudes of P3a at CPz were significantly greater than those at Pz (*P* < 0.001).

**FIGURE 2:**
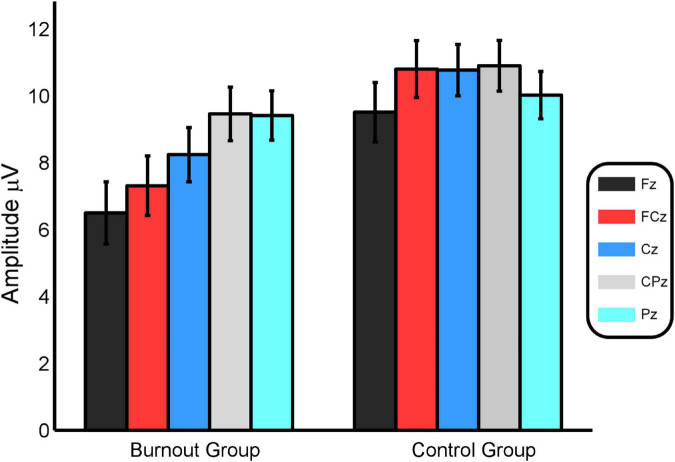
A comparison of the average P3a amplitudes at the five electrode sites between the burnout group and the control group.

At Fz, FCz, and Cz, the average P3a amplitudes of the burnout group were significantly smaller than those of the control group (*P* = 0.024, *P* = 0.007, *P* = 0.029, respectively). No significant difference in the average P3a amplitude was identified between the two groups at CPz and Pz (*P* = 0.200, *P* = 0.557). See Figure 2.

**FIGURE 3:**
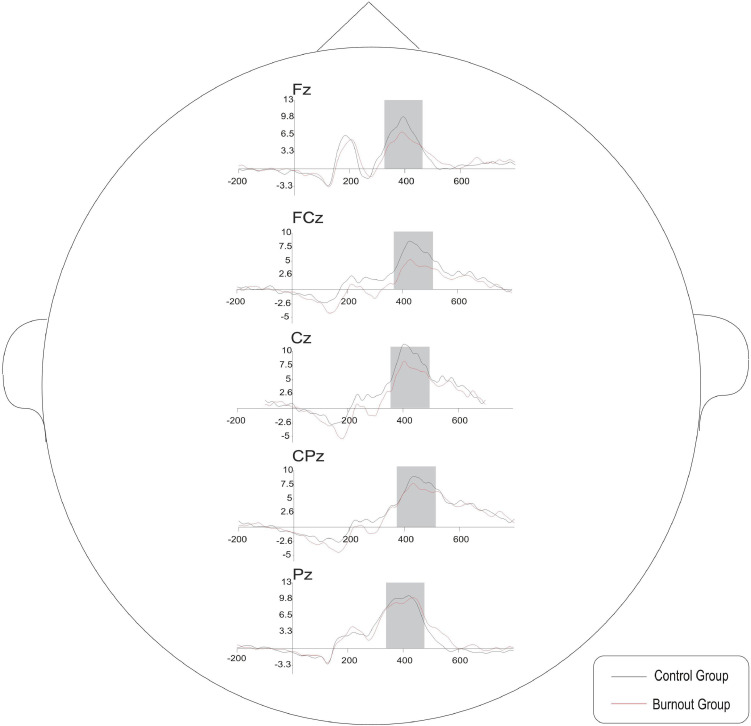
The average P3a waveforms of the five electrode sites in the burnout group and control group in the oddball paradigm.

**FIGURE 4:**
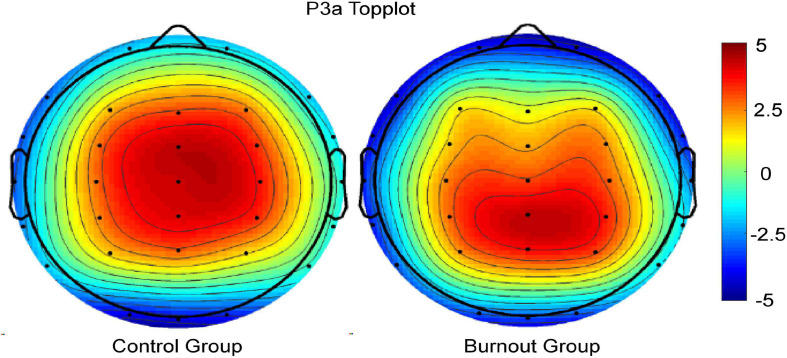
Brain topographic map of P3a in the burnout group and the control group.

The average waveforms of the five electrode sites, Fz, FCz, Cz, CPz, and Pz, and the brain topography of P3a are shown in Figures 3, 4.

### Comparison of the average amplitude of P3b between the burnout group and control group

The descriptive statistics for the P3b average amplitude at each electrode site in the burnout group and control group are shown in Table 5.

**TABLE 5:** Description of the average P3b amplitude in the burnout and control groups ($\bar{\text{x}}$ ± SE, μV).

Electrode site	Burnout group (*n* = 19)	Control group (*n* = 21)
Fz	3.44 ± 1.04	6.72 ± 0.99
FCz	4.11 ± 1.06	8.54 ± 1.01
Cz	5.24 ± 1.05	8.72 ± 1.00
CPz	6.91 ± 0.95	8.96 ± 0.90
Pz	7.53 ± 0.95	8.49 ± 0.91

The repeated-measures ANOVA results indicated that the main effect was significant between groups, *F*(1, 38) = 4.645, *P* = 0.038, η^2^ = 0.109, and that the P3b average amplitude of the burnout group was significantly lower than that of the control group, i.e., 5.45 ± 0.96 and 8.29 ± 0.91 μV, respectively.

The main effect of the electrode was significant, *F*(4, 152) = 22.771, *P* < 0.001, η^2^ = 0.375, and the average amplitude of P3b induced at the electrode sites in the parietal region was greater than that in the prefrontal region and the central parietal region. The average amplitudes of P3b at Fz, FCz, Cz, CPz, and Pz were 5.08 ± 0.72, 6.33 ± 0.73, 6.98 ± 0.72, 7.94 ± 0.65, and 8.01 ± 0.66 μV, respectively.

The interaction effect between electrode and group was significant, *F*(4, 152) = 6.961, *P* = 0.005, η^2^ = 0.155. Further simple effect analysis indicated that in the burnout group, the average amplitude of P3b at Fz was smaller than that at Cz, CPz, and Pz (Fz < Cz < CPz < Pz), with significant differences (*P* = 0.001, *P* < 0.001, *P* < 0.001); the average amplitude of P3b at FCz was smaller than that at Cz, CPz, and Pz (FCz < Cz < CPz < Pz), with significant differences (*P* < 0.001, *P* < 0.001, *P* < 0.001); the average amplitude of P3b at Cz was smaller than that at CPz and Pz (Cz < CPz < Pz), with significant differences (*P* < 0.001, *P* < 0.001); the average amplitude of P3b at CPz was smaller than that at Pz (*P* = 0.011); and the average amplitude of P3b at Fz and FCz was not significantly different (*P* = 0.064). In the control group, the average amplitude of P3b at Fz was smaller than that at FCz, Cz, CPz, and Pz (Fz < FCz < Cz < CPz < Pz), with significant differences (*P* < 0.001, *P* < 0.001, *P* = 0.002, *P* = 0.028); the average amplitude of P3b at FCz was not significantly different from that at Cz, CPz, and Pz (*P* = 0.518, *P* = 0.420, *P* = 0.940); the average amplitude of P3b at Cz was not significantly different from that at CPz and Pz (*P* = 0.425, *P* = 0.634); and the average amplitude of P3b at CPz was greater than that at Pz (*P* = 0.041).

**FIGURE 5:**
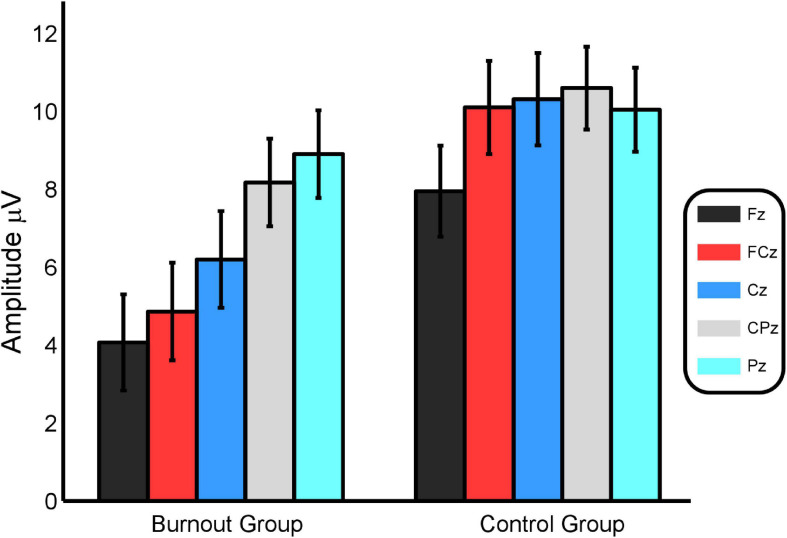
A comparison of the average P3b amplitudes at the five electrode sites between the burnout group and the control group.

At Fz, FCz, and Cz, the P3b average amplitude of the burnout group was significantly smaller than that of the control group (*P* = 0.028, *P* = 0.004, *P* = 0.021). No significant difference in the average P3b amplitude was found between the two groups at CPz and Pz (*P* = 0.124, *P* = 0.469). See Figure 5.

**FIGURE 6:**
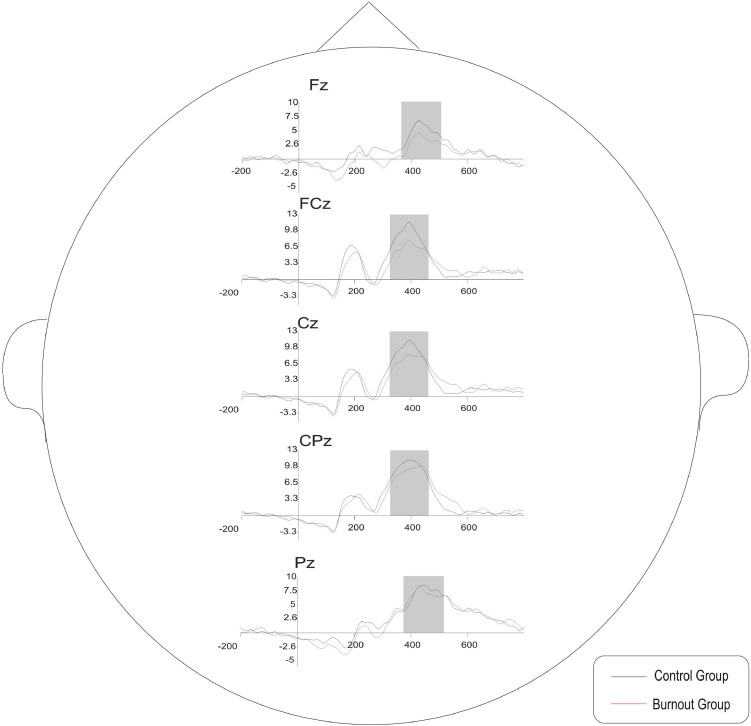
The average P3b waveforms of the five electrode sites in the burnout group and control group in the oddball paradigm.

**FIGURE 7:**
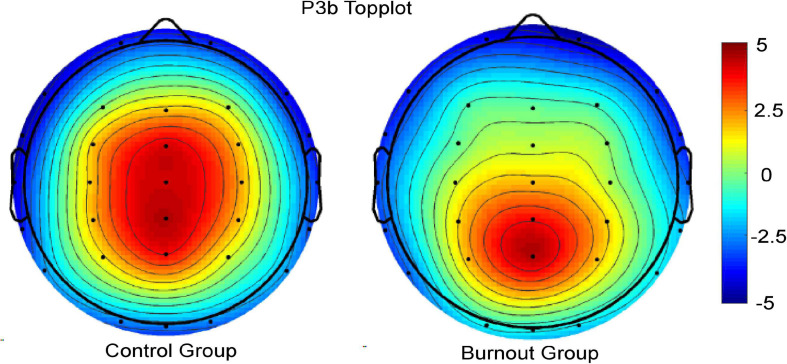
Brain topographic map of P3b in the burnout group and control group.

The average waveforms of the five electrode sites, Fz, FCz, Cz, CPz, and Pz, and the brain topography of P3b are shown in Figures 6, 7.

## Discussion

The present study investigated the job burnout of army soldiers and officers, and examined the effect of job burnout on the attention ability of them using ERP. The results mainly included two aspects. First, the epidemiological survey of job burnout showed that there was a certain degree of job burnout in army individuals, and the incidence was about 30%. Second, compared with the control group, the participants in the burnout group showed lower amplitudes of P3a and P3b significantly at the five electrode points on the midline of the brain, but there was no significant difference in the numbering of target stimuli in the Oddball task.

### Military burnout results

As a special group compared with civilians, military soldiers and officers have different missions, working environments, and management styles. Most recent research focuses on military job burnout caused by wartime stress, with relatively insufficient attention toward systematic discussions of military job burnout during peacetime (Vinokur et al., 2011). In this study, using the Job Burnout Scale for Military Personnel, the average burnout score for soldiers and officers was 0.74 ± 0.46, which was lower than the scores of the Xinjiang land force and air force personnel reported by Xinzhen Meng but higher than those of the Xinjiang armed police (Meng et al., 2013). The scores in this study are similar to the overall score for troops stationed in Xinjiang (Su et al., 2015). In this study, 317 soldiers and officers had average job burnout scores ≥1, accounting for 29.85% of the total participants, which is lower than the detection rate of job burnout for sergeants in the armed forces assessed using the Chinese job burnout scale (Hao, 2019). Since the objects of the Job Burnout Scale for Military Personnel are the military individuals and the measurement of the public burnout mainly uses Maslach Burnout Inventory (MBI), we have not been able to directly obtain the comparison of the degree and detection rate of burnout between this sample and the public. According to the existing literature, even in times of peace, military individuals also report higher acute/chronic stress, fatigue, stress, depression, anxiety, somatization, etc. compared with ordinary citizens as a result of task specificity, deployment systems in arduous military bases, and strict and highly organized management (Martins and Lopes, 2012; Yang et al., 2016; Straud et al., 2019; Blais et al., 2021). The factors and phenomenon mentioned above may lead to a high detection rate and severity of job burnout. The reasons for the high burnout scores for army troops are as follows: these personnel, especially those responsible for frontier defenses or patrolling snowy plateaus, deserts, and neutral zones, have poor working and living environments, engage in extensive combat readiness tasks and military training, and lack cultural activities and external communication. Long-term chronic stress undoubtedly aggravates the psychological pressure experienced by soldiers and officers, leading to a lack of enthusiasm, boredom, fatigue, resistance to management and a high level of job burnout (Martins and Lopes, 2012; Mota et al., 2012). Notably, the interpersonal relationships between individuals are relatively good because the score for the interpersonal relationship dimension was the lowest, indicating that soldiers and officers can effectively use social support among friends to cope with adverse conditions. Improving social support is considered to be an effective strategy to reduce stress and job burnout (Hamaideh, 2011, 2012).

### Behavioral results

The behavioral results showed that, compared with the control group, the counting of target stimuli in the burnout group had shown a downward tendency, but no significant difference was found. In our study, participants only needed to answer the counting of target stimuli orally after completing the task, and there was no need to make any key-press response after each target stimuli appears. That is to say, there is no trade-off between speed and accuracy in this task, which is different from the task transformation paradigm used by Sokka et al. (2017). Previous behavioral researches show impaired performance in the domains of attention and executive functions in severe burnout, as indicated by slower reaction time (Oosterholt et al., 2014; Kleinsorge and Scheil, 2015), higher error rates (Diestel et al., 2013), or both (Sandström et al., 2005). In the absence of time pressure (as opposed to the need to respond quickly to a button), the behavior performance of the Oddball task in this study still has a downward tendency. We speculate that burnout may have led to insufficient processing of cognitive tasks. The attention function may be damaged. However, no significant difference was found in the behavioral performance results, which may be related to task difficulty, brain compensation effect, and sample size. This will be discussed in more detail later.

### Event-related potential results

In this study, P3a and P3b in the burnout group were lower to varying degrees not only in the overall average amplitude but also at the Fz, FCz, Cz, CPz, and Pz electrode points than that in the control group. Our findings are consistent with most existing research on the P300 associated with job burnout (Gajewski et al., 2017b; Golonka et al., 2018). Van Luijtelaar et al. (2010) linked lower P300 in the burnout group with attention and memory defects. Golonka et al. (2018) reported that reduced P300 reflected limited attention resources and may be considered as an indicator of workload and/or fatigue in the population. It is generally agreed that the amplitude of ERP is positively correlated with the input of psychological resources, with a smaller amplitude corresponding to a lower amount of psychological resources invested (Helfrich and Knight, 2019; Wu et al., 2019). It can be inferred that job burnout has led to a lower amount of psychological resources invested by soldiers and officers. Here, the amount of psychological resources invested may represent two aspects: one is the absolute insufficiency of the total amount of attention resources, and the other is the insufficient conversion and allocation of attention resources between different stimuli (Van Luijtelaar et al., 2010; Giraudet et al., 2015). When there is no or mild burnout and low task load, both target and novel stimuli can be processed, and novel stimuli are more likely to be detected (Lavie et al., 2004; Lavie, 2005). When the task load and job burnout increase, attentional resources must be allocated to different stimuli, giving priority to task-related or interested stimuli and processing. However, when severe burnout happens and the task load exceeds the range that cognitive resources can handle, the processing of target and novel stimuli can be impaired. In this study, the significantly lower amplitudes of P3a and P3b in the burnout group represent the lower ability to deal with target and novel stimuli, which also confirms the results and views of Sokka et al. (2016). The difference is that Sokka et al. used auditory sound as novel stimuli while we used visual graph, but this also precisely indicates that the attention function impairment caused by burnout is cross-sensory channel, which is jointly manifested as the reduction of P3a and P3b amplitudes in neurophysiological indicators. The counting of the target stimuli is the task requirement, it has been informed to the participants and the shape of target stimuli is specified before the start. In this process, participants will combine their current behavioral goals and previous experience to represent the given stimuli in advance, and even expect the target stimuli to appear, which requires participants to actively and diligently call attention resources. The prefrontal cortex integrates the characteristics and then forms priority maps, causing attention to focus on the task goal. In this task, participants were not informed about novel stimuli in advance, they had no knowledge of the emergence or shape of the novel stimuli. When novel stimuli with sufficient intensity appear, the participants unconsciously produce passive directional responses. Because no reaction is necessary or related to the task, willpower and effort are not required. This is consistent with existing views and is also the characteristic of top-down voluntary attention and bottom-up involuntary attention (Friedman et al., 2001; Polich and Criado, 2006; Katsuki and Constantinidis, 2014). Although the traditional theory holds the views that the frontal lobe is the dominant brain area of P3a, the parietal lobe is the dominant brain area of P3b, this study did not show the most typical characteristics and completely corresponding relationships between P3a, P3b, and the brain areas mentioned above. We observed both the brain topographies of P3a and P3b have a central-frontal peak, and we believe that this may be caused by the conduction of cognitive neural activities. The cognitive activity model of P300 proposed by Schreiner and Staudigl (2020) also provides evidence for our view. When stimuli occur, they are first captured by sensory channels and then enter attention and working memory, activating the frontal lobe to generate P3a. Subsequently, the brain processes, encodes, and stores the stimuli, and the temporal parietal lobe is activated, updating the memory to generate P3b (Blair, 2017). Polich (2007) found that the anterior cingulate cortex (ACC) regulates the process of P3a transmission from the frontal to the temporoparietal cortex to generate P3b. In addition, the activation process from the frontal cortex to the temporoparietal lobe has been demonstrated by brain imaging (Gudi-Mindermann et al., 2020). We believe that when different types of stimuli occur randomly and sequentially, the brain processes, encodes, and stores previous stimuli, while sensory input of new stimuli is performed. These cognitive activities are carried out simultaneously or alternately rather than independently. The superposition of different stimulus types and different cognitive processes reveals the complexity of the brain’s high-level neural activities. The process from being perceived to processed has been carried out simultaneously or alternately of target and novel stimuli, and the activation and conduction from frontal lobe to parietal lobe are also occurring synchronously, which may lead to the above phenomenon. It may also be caused by certain limitations in the selection of ERP technology and indicators. If possible, in the future, we will conduct in-depth studies in specific brain areas in combination with intracranial EEG, high-resolution magnetic resonance, and positron emission computed tomography (PET).

In addition, through the item analysis of the Job Burnout Scale for Military Personnel, we believe that the impact of burnout on the voluntary and involuntary attention may not be directly generated. Burnout may damage the attention function by causing stress, emotional disorders, somatization symptoms, etc. The amplitude of P300 has also been found to become lower in association with high stress (Shackman et al., 2011), increased sleepiness (Polich and Kok, 1995; Colrain and Campbell, 2007), depression (Cavanagh and Geisler, 2006), and low arousal level (Polich and Kok, 1995; Colrain and Campbell, 2007; Shackman et al., 2011). Some studies have reported that stress impairs top-down attention control (Plessow et al., 2012a,b) and leads to distractibility of attention (Sänger et al., 2014). In the Job Burnout Scale for Military Personnel, item 2 “Relieve work pressure by smoking and drinking,” item 6 “I have full confidence in my work,” and item 12 “Avoid contact with colleagues deliberately” describe performance under pressure from work and social stress. As a psychosomatic exhaustion syndrome, job burnout may induce more intense emotional reactions in a given task (Gray et al., 2004). It also has negative impacts on the sensitivity of sensory channels, different functional areas of the brain, and the neurophysiological connections among them, impairing attention ability directly or indirectly (Sokka et al., 2017). Item 16 “Not happy” and item 21 “Work makes me nervous and anxious” in the scale assessed the emotional impact caused by burnout. Similarly, somatization symptoms caused by burnout would also impair attentional function (Hall et al., 2011), such as item 3 “I have a headache when having a heavy workload” and item 9 “feel weak.” We may have had that experience, when we feel stressed, exhausted, depressed, anxious, and accompanied by headache, sleepiness, etc., at work, we are unlikely to be able to concentrate the focus on one thing, and at the same time, sudden stimuli or events are also difficult to arouse our high attention level.

### Compensation effect of cognitive resources

It is worth noting that, although the amplitudes of P3a and P3b in the burnout group were lower than that in the control group significantly, there was no significant difference in behavioral performance. The following reasons may contribute to the phenomenon above. First, the Oddball task may be easy for military personnel within that age range because the average numbers counted were close to the real numbers of target stimuli, and no significant differences were found in either group regardless of whether job burnout was present. That is, the task was sufficiently simple for all participants, and they had adequate cognitive resources to complete the task. However, this reason is not sufficient to explain the ERP characteristics of the burnout group. If the task was sufficiently simple and did not consume cognitive resources for the individuals in the burnout group, their P3a and P3b amplitudes should not become lower significantly. Second, the difficulty of the task may have damaged the cognitive function of the participants, which was manifested by the lower amplitudes of P3a and P3b and the number of target stimuli counted in the burnout group. However, to maintain high performance, burnout participants tried to invoke other resources to compensate for the damaged cognitive resources. We believe that this is a compensation effect of cognitive resources, that is, people who have exhausted cognitive resources in some cognitive fields may invest more resources in other fields and additional compensatory efforts to compensate for performance deficits such as accuracy and reaction time and to prevent errors (O’Connell et al., 2012; Saliasi et al., 2013). The same results appear in the subclinical burnout individuals and elderly individuals (they are thought to have impaired attention and working memory) (Schneider-Garces et al., 2010; Sokka et al., 2016; Morrison and Taler, 2020) as well as in different cognitive tasks (two-back task, flanker task, matching task, and digit span task) (Oosterholt et al., 2014). However, the mechanism of brain compensation effect and what kind of resources are used for compensation have not been clarified. This phenomenon and mechanism will be investigated in depth in future research.

### Application in military affairs

The existing research on job burnout among front-line military personnel is very limited. To a certain extent, this study fills the gap in this field and provides valuable contributions. For military missions, soldiers and officers must actively invest attention resources to identify and track targets in complex environments. Additionally, they must also recognize sudden situations involving enemies and be aware of battlefield situations to adjust their actions and deployment. Therefore, the voluntary and involuntary attention abilities of military personnel play important roles in their combat effectiveness (Heaton et al., 2014). The results from this study indicate that a certain degree of job burnout occurs among army soldiers and officers. Voluntary attention and involuntary attention are damaged to a certain extent, resulting in not only a lack of focus on current military missions but also decreased awareness of dangerous situations, which seriously affects combat effectiveness. In the military field, the selection and training of military personnel are two important aspects. How to effectively and accurately select fighters who can adapt to different posts and how to improve the mental health and task performance of soldiers and officers are worth considering. By analyzing the results of this study, we believe that P300 (P3a and P3b) can be used as markers capable of predicting the susceptibility to burnout before it occurs. In the later stage, different characteristic indicators of the ERP process can be extracted by machine learning and entered into the classifier to establish a database of burnout in different populations. Early screening for burnout in the future provides an adjunctive diagnosis. Soldiers and officers can be objectively evaluated during daily training to monitor and identify job burnout in the early stage. This study also provides ideas for interventions; that is, psychological training and interventions can be carried out to improve voluntary and involuntary attention, at the same time, P3a and P3b can be used as evaluation indicators of training and intervention effects to counteract the damage in attention caused by job burnout and improve military performance.

### Limitations and prospects

The present study has several limitations and prospects. First, the participants selected in this study completed the Job Burnout Scale for Military Personnel, which is a highly subjective self-evaluation burnout measurement method. In a follow-up study, objective evaluations and clinical diagnoses may strengthen the results observed in this study. Second, Huibers et al. (2003) studied the relationship among persistent fatigue, burnout, and chronic fatigue syndrome and found that some characteristics are shared among these conditions. Therefore, comorbid diseases or symptoms such as burnout, chronic fatigue syndrome, and depression should be considered (Van Dam, 2016). Third, some scholars (Diestel et al., 2013) believe that when a task is associated with high executive control requirements, significant differences will be evident between groups with different levels of burnout; that is, individuals with burnout should be distinguished only by analyzing specific conditions and functions. The specific conditions of participants, such as engagement, emotion, and arousal status, should also be analyzed. Fourth, we can change the task load by changing the proportion of target stimuli and novel stimuli in the Oddball task, measure P300 and task performance (such as response time, accuracy, and other external indicators) to explore the characteristics of neuroelectrophysiological and behavioral indicators for revealing cognitive impairment under different task loads. Fifth, as Kappenman and Luck (2010) fully proved the detrimental effects of increasing the high pass filter setting on P3 amplitude and the entire ERP waveform, and many studies have set the high pass below 0.1 Hz, the results using 1 Hz high-pass filtering in this study considered by experimental conditions and experience have some limitations. In the future, it is better to set a more reasonable filtering range based on actual research settings and experience. Sixth, in a follow-up study, psychological or behavioral interventions can be applied and then evaluated using pretests and posttests with the goal of selecting and implementing effective methods to alleviate military job burnout.

## Conclusion

In this study, ERP was used to study the attention ability of army soldiers and officers with job burnout. A certain degree of job burnout was evident among army soldiers and officers, and the voluntary and involuntary attention abilities of individuals with burnout were damaged to some extent, as reflected by the lower amplitudes of P3a and P3b. The results suggest that P3a and P3b can be used as indicators to monitor cognitive neural function in individuals with burnout. P3a and P3b can also be used as references for evaluating the effects of cognitive training and screening methods. It is expected to be applied in the military field in the future.

## Data availability statement

The raw data supporting the conclusions of this article will be made available by the authors upon reasonable request.

## Ethics statement

The studies involving human participants were reviewed and approved by the Clinical Trial Ethics Committee of the First Affiliated Hospital of Air Force Medical University and is registered at the Chinese Clinical Trial Registry (CHiCTR1800019761). The patients/participants provided their written informed consent to participate in this study.

## Author contributions

LWu and YC were responsible for the writing of this manuscript. XuL, PF, WL, HL, XiL, YL, LWa, CL, and TZ were responsible for the experimental collection of original data. LWu, YC, TF, KS, and LR were responsible for the data collection and analysis. SW and CN were responsible for the experimental design and overall planning of the research. All authors read and approved the final manuscript.
